# Transcriptomic Profiles of Long Noncoding RNAs and Their Target Protein-Coding Genes Reveals Speciation Adaptation on the Qinghai-Xizang (Tibet) Plateau in *Orinus*

**DOI:** 10.3390/biology13050349

**Published:** 2024-05-16

**Authors:** Qinyue Min, Kaifeng Zheng, Tao Liu, Zitao Wang, Xiuhua Xue, Wanjie Li, Yuping Liu, Yanfen Zhang, Feng Qiao, Jinyuan Chen, Xu Su, Shengcheng Han

**Affiliations:** 1 Key Laboratory of Biodiversity Formation Mechanism and Comprehensive Utilization of the Qinghai-Tibet Plateau in Qinghai Province, School of Life Sciences, Qinghai Normal University, Xining 810008, China; m1nq1nyue@163.com (Q.M.); 18587712819@163.com (Z.W.); lyp8527970@126.com (Y.L.); 13897450796@163.com (Y.Z.); qiaofnm@163.com (F.Q.); 20211027@qhnu.edu.cn (J.C.); 2 Beijing Key Laboratory of Gene Resources and Molecular Development, College of Life Sciences, Beijing Normal University, Beijing 100875, China; kaifeng_zheng@mail.bnu.edu.cn (K.Z.); xiuhuaxue@bnu.edu.cn (X.X.); lwj@bnu.edu.cn (W.L.); 3 School of Ecology and Environmental Science, Qinghai University of Science and Technology, Xining 810016, China; lt532226527@gmail.com; 4 Academy of Plateau Science and Sustainability, Qinghai Normal University, Xining 810008, China

**Keywords:** long noncoding RNA, protein-coding genes, speciation adaptation, *Orinus*

## Abstract

**Simple Summary:**

*Orinus*, a genus distributed in alpine regions of the Qinghai-Xizang (Tibet) Plateau (QTP), includes the following two species: *O. thoroldii*, predominantly in the western QTP at elevations between 3300 and 4700 m, and *O. kokonoricus*, in the eastern QTP at relatively low altitude between 2500 and 3400 m. However, the mechanism of long noncoding RNA (lncRNA)-regulated speciation adaptation to high altitudes and cold arid environments in *Orinus* remains unclear. In this study, we first identified the transcriptome-wide features of lncRNAs in *O. thoroldii* and *O. kokonoricus,* including five main types of lncRNAs that class_code with “i, o, u, x, j”. Then, an evolutionary tree was conducted to implicate the relationships among 39 species with the count of conserved lncRNAs. Moreover, we sought to reveal the mechanism of cis-regulation from differentially expressed lncRNAs (DElncRNAs) and their nearby protein-coding genes (PCGs) between *O. thoroldii* and *O. kokonoricus*. GO analysis showed that DElncRNAs and their potential target PCGs participate in different biological processes between *O. thoroldii* and *O. kokonoricus*, indicating their speciation adaptation on the QTP. In addition, the specifically expressed transcription factors (TFs) in *O. thoroldii* suggested that DElncRNAs play a vital role in adapting to the environment through the expressed regulation of TFs.

**Abstract:**

Long noncoding RNAs (lncRNAs) are RNA molecules longer than 200 nt, which lack the ability to encode proteins and are involved in multifarious growth, development, and regulatory processes in plants and mammals. However, the environmental-regulated expression profiles of lncRNAs in *Orinus* that may associated with their adaptation on the Qinghai-Xizang (Tibet) Plateau (QTP) have never been characterized. Here, we utilized transcriptomic sequencing data of two *Orinus* species (*O. thoroldii* and *O. kokonoricus*) to identify 1624 lncRNAs, including 1119 intergenic lncRNAs, 200 antisense lncRNAs, five intronic lncRNAs, and 300 sense lncRNAs. In addition, the evolutionary relationships of *Orinus* lncRNAs showed limited sequence conservation among 39 species, which implied that *Orinus*-specific lncRNAs contribute to speciation adaptation evolution. Furthermore, considering the *cis*-regulation mechanism, from 286 differentially expressed lncRNAs (DElncRNAs) and their nearby protein coding genes (PCGs) between *O. thoroldii* and *O. kokonoricus*, 128 lncRNA-PCG pairs were obtained in *O. thoroldii*, whereas 92 lncRNA-PCG pairs were obtained in *O. kokonoricus*. In addition, a total of 19 lncRNA-PCG pairs in *O. thoroldii* and 14 lncRNA-PCG pairs in *O. kokonoricus* were found to participate in different biological processes, indicating that the different expression profiles of DElncRNAs between *O. thoroldii* and *O. kokonoricus* were associated with their adaptation at different elevations on the QTP. We also found several pairs of DElncRNA nearby transcription factors (TFs), indicating that these DElncRNAs regulate the expression of TFs to aid *O. thoroldii* in adapting to the environment. Therefore, this work systematically identified a series of lncRNAs in *Orinus*, laying the groundwork for further exploration into the biological function of *Orinus* in environmental adaptation.

## 1. Introduction

Long noncoding RNAs (lncRNAs) are the genes that are transcribed to lengths exceeding 200 nt without encoding proteins [1]. In the past decade, noncoding elements located in intergenic regions including lncRNA were considered as junk sequences occupying a large area in the genome. With the deepening of research, it has been discovered that over 90% of the eukaryotic genome is capable of transcription, leading to the production of huge lncRNAs [2,3]. The majority of lncRNAs contain conserved promoters and are transcribed by RNA polymerase II [4]. Based on the relative position with protein-coding genes in the genome, lncRNAs can be categorized into several primary types as follows: intergenic type, antisense type, intron type, and potentially novel type [5]. LncRNA was first reported in humans, and then many lncRNAs were characterized in mammals with important functions to date [6,7,8]. However, only a few lncRNAs with distinct functions have been studied, and most of the research has focused on the prediction of lncRNA function and identification, including aspects involved in growth and development processes, metabolic processes, and responses to diverse hormones and stresses in plants [9,10,11,12]. The two vernalization-induced lncRNAs, i.e., *COOLAIR* [13] and *COLDAIR* [14], have the ability to inhibit the expression of *FLOWERING LOCUS C* (*FLC*) through epigenetic silencing, leading to the initiation of flowering in *Arabidopsis* [15]. With the in-depth investigation of lncRNAs, multiple research efforts have revealed the participation of lncRNAs in responding to extreme habitats. Qin et al. [16] identified a lncRNA named *DROUGHT-INDUCED lncRNA (DRIR)* in *Arabidopsis*, which functions as a positive regulator in responding to drought and salt stress. While *DRIR* had a low expression level in normal conditions, its activity increased significantly when exposed to abiotic stress and abscisic acid (ABA) treatment. In *Arabidopsis*, lncRNA *HSFB2a* was initially found to be related to heat stress adaption. Wunderlich et al. [17] showed that *HSFB2a* was needed in the development of female gametophytes. In hexaploidy wheat, lncRNA *VAS* (*TaVRN1* alternative splicing), was shown to induce earlier flowering after over-expression [18]. These results indicated that lncRNAs could play key roles in plant adaption to the environment. 

The Qinghai-Xizang (Tibet) Plateau (QTP) is the largest plateau in China and also the highest plateau in the world. In addition, the QTP has the largest permafrost area and has a unique geographical location. Its overall climate is characterized by substantial temperature variance, strong ultraviolet radiation, high sunshine, and so on [19]. The QTP is rich in plant and animal resources. To survive in this region, organisms have formed a unique adaptation mechanism in stressful environments, such as low temperatures and droughts, through natural selection for a long time, which provides good materials for the study of biological adaptability on the plateau [20]. *Orinus* belongs to the grass family (Poaceae) and is a genus found in alpine regions that is known for its significant economic value [21]. There are two species of *Orinus* including *Orinus thoroldii* (Stapf ex Hemsl.), which is predominantly distributed in the western QTP at elevations between 3300 and 4700 m, and *Orinus kokonoricus* (K. S. Hao), which occurs in the eastern QTP at a relatively low altitude between 2500 m and 3400 m [22]. Both of them play an important role as valuable forage resources and are highly nutritious for local livestock in arid regions. The main distinction between the two species of *Orinus* lies in their altitudinal distributions [23]. In the QTP, *Orinus* contributes to soil stabilization, which has ecological and conservational significance, particularly attributed to the prolific root system; therefore, this genus could serve as a valuable model for revealing the timing and mechanisms of desertification in the QTP [22]. In recent years, there have been many reports on the adaptation of Poaceae to extreme habitats including low temperature and drought conditions both domestically and internationally [24,25,26]. However, research on the mechanism of *Orinus’* adaptation to high altitudes and cold arid environments remains elusive. Therefore, systematically analyzing the molecular mechanism of *Orinus’* adaptation to high altitudes and high cold and arid environments can enhance our comprehension of the genus’ origins and bolster conservation initiatives for extreme conditions.

In this work, we focused on two typical samples of *O. thoroldii* and *O. kokonoricus* from the west and east of QTP at different altitudes, separately. Using previously published RNA-seq data [27], we conducted systematic lncRNA identification for investigating the expression patterns of lncRNAs between the two species of *Orinus*, characterized the transcriptomic profiles of lncRNAs in them, and predicted the function of DElncRNAs dependent on the expression pattern of their nearby PCGs. All in all, this work not only characterized a large number of lncRNAs in *Orinus* but also provided preliminary insights about the molecular mechanisms by which lncRNAs regulate the adaptation of *Orinus* to extreme habitats in the QTP.

## 2. Materials and Methods

### 2.1. Plant Samples

Two species of *Orinus* were chosen; one was *O. thoroldii*, obtained from Gongga country in Xizang (29°0′27.0″ N, 85°26′48.8″ E; alt. 4687 m), and the other was *O. kokonoricus*, obtained from Gonghe country in Qinghai (36°21′26.3″ N; 100°43′5.8″ E; alt. 3130 m) [27]. The plant samples were obtained from individuals thriving in their natural environments in typical circumstances. Su et al. [27] provided additional details on growth conditions and transcriptome sequencing information.

### 2.2. Overall Transcriptome Mapping and Assembly

To obtain the paired-end RNA-seq information for the two species of *Orinus*, we used the NCBI Sequence Read Archive (Accession: PRJNA385721) and extracted the data for *O. thoroldii* and *O. kokonoricus* [27]. The detailed computational pipeline for systematic analysis based on transcriptome sequencing data is presented. We used Fastp (version 0.23.4; https://github.com/OpenGene/fastp, accessed on 18 October 2023; -q 30 -l 20) to evaluate the quality of the original reads and refine the raw data through trimming. In this study, we selected the *Orinus kokonoricus* genome (assembly: PRJCA018722) as the reference genome [23]. We applied Bowtie2 (version 2.5.1; https://github.com/BenLangmead/bowtie2, accessed on 11 December 2023) and TopHat2 (version 2.1.1; https://github.com/DaehwanKimLab/tophat, accessed on 12 December 2023; -I 5000) for the construction of the genome index and alignment of reads. For the assembly and merging of transcripts, Cufflinks (version 2.1.1; https://anaconda.org/bioconda/cufflinks, accessed on 13 December 2023) was utilized, and fragments per kilobase of transcript per million mapped fragments (FPKM) was used to standard the expression levels of isoforms or genes. 

### 2.3. Pipeline for lncRNA Identification

**Figure 1:**
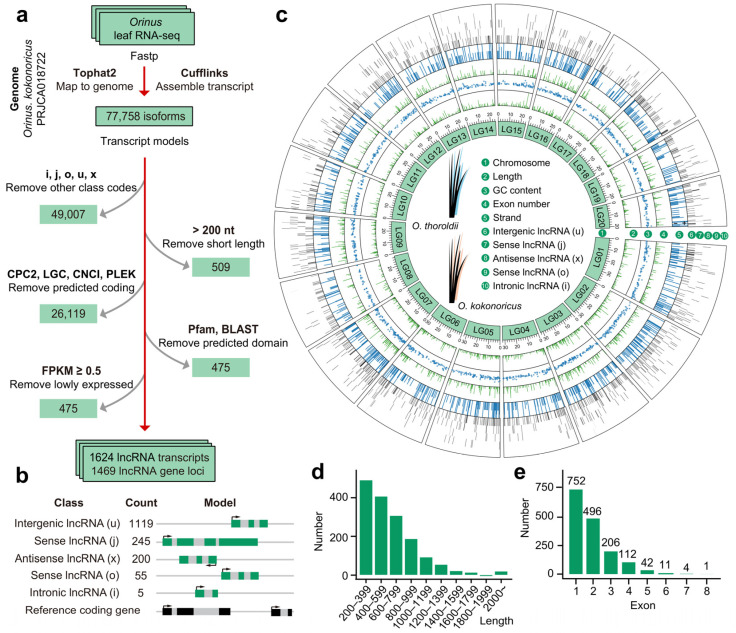
Comprehensive profiling and characterization of long non-coding RNAs (lncRNAs) across the transcriptome of *Orinus*. (**a**) Schematic computational pipeline for the identification of lncRNAs in *Orinus*. We removed other class code types and kept the following five kinds. “i”: transcripts falling entirely within a reference intro. “o”: generic exonic overlap with a reference transcript. “u”: intergenic transcripts. “j”: potentially novel isoforms. “x”: natural antisense transcript (http://cole-trapnell-lab.github.io/cufflinks/cuffcompare/). (**b**) Classification of *Orinus* lncRNAs according to the positional relationship between the transcripts and annotated genes in the reference assembly. (**c**) Transcriptome-wide characterization of *Orinus* lncRNAs. “1”: basic skeleton and relative sizes of the 20 *Orinus* chromosomes. “2”: lower length distribution characteristics of lncRNAs in *Orinus*. “3”: fluctuations in the GC content of lncRNAs that rarely have a very high or extremely low GC content. “4”: fluctuations in the number of lncRNA exons. “5”: lncRNAs with defined strand sense (+) or antisense (−). “6”–“10”: relative positions of all lncRNAs (five types) on the 20 chromosomes. (**d**) Distribution of lncRNA lengths in *Orinus*. (**e**) Exon number distribution among *Orinus* lncRNAs.

To characterize the biological properties of lncRNAs, we developed a meticulous screening pipeline (Figure 1a). Transcript models with “i, o, u, x, j” were isolated and progressed through the subsequent filtering process (https://anaconda.org/bioconda/cufflinks/cuffcompare/, 13 December 2023). To ensure the qualified length of lncRNAs, we removed transcripts with nucleic acid sequences less than 200 nt. Coding Potential Calculator (CPC2 version 1.0.1), LGC (version 1.0), Coding-Noncoding Index (CNCI version 2), and PLEK (version 1.2) were utilized to predict the coding potential of the transcript [28,29,30,31]. In addition, the pfam database and BLASTP were used to analyze and filter potential protein domains in the transcript sequences (E-value < 1 × 10^−5^) [32,33]. Additionally, potential miRNA precursors in the transcripts were removed based on mature miRNA sequences from miRbase (https://www.mirbase.org/, accessed on 13 December 2023) [34]. Finally, the transcripts were filtered according to the expression level, namely, fragments per kilobase of transcript per million mapped reads (FPKM) < 0.5. To illustrate the comprehensive characteristics of lncRNAs in *Orinus*, a circos plot was created using TBtools [35]. Thirty-nine different species of lncRNAs were used to conduct the homologous analysis, and *O. kokonoricus* was the reference species. Using the CANTATA database (http://rhesus.amu.edu.pl/CANTATA/index.html, accessed on 14 December 2023) and PLncDB (https://www.tobaccodb.org/plncdb/Download, accessed on 14 December 2023), lncRNAs with E-values < 10^−5^ were considered conserved in our analysis. It should be noted that all the plots and figures (such as flowcharts, circos diagrams, heat maps, and histograms) were generated in Prism (version 8.0.2), TBtools (version 2.088), and Adobe Illustrator 2023 (version 27.0). 

### 2.4. Differential Expression Analysis

We used Cuffdiff to analyze transcript expression differences. Significant differentially expressed lncRNAs (DElncRNAs) in the two species of *Orinus* were determined based on FPKM values. We used TBtools to illustrate the intersections in the two species of *Orinus* by creating a Venn diagram. Here, we characterized significant differentially expressed based on the criterion of |log2 (fold change)| values ≥ 1 and *p*-value ≤ 0.01.

### 2.5. Prediction of Cis-Regulated Target Genes of DElncRNAs

Target genes predicted to be *cis*-regulated were those transcribed upstream or downstream of DElncRNAs within a 100 kb window [36]. Using Bedtools (version 2.30.0; https://github.com/arq5x/bedtools2/releases, accessed on 14 December 2023), we extracted all PCGs located around the DElncRNAs in the two species of *Orinus*. 

### 2.6. Gene Ontology Enrichment Analysis 

Our main emphasis was on investigating the potential biochemical effects of the PCGs of *Orinus* DElncRNAs by employing the eggNOG-mapper tool (http://eggnog-mapper.embl.de/, accessed on 14 December 2023). We conducted a Gene Ontology (GO) analysis of the protein sequences derived from these target protein-coding genes [37].

### 2.7. Transcription Factor Survey

The protein-coding genes neighboring the *Orinus* DElncRNAs were analyzed to identify potential transcription factors using PlantTFDB [38]. 

## 3. Results

### 3.1. LncRNA Screening Lays the Foundation for the Further Understanding of the Molecular and Biological Functions of Non-Coding Genes in Orinus

To carry out a comprehensive exploration of long noncoding RNAs in *Orinus*, we followed the lncRNA screening pipeline to filter transcript models in the leaf transcriptome of *O. thoroldii* and *O. kokonoricus* [27]. Based on the latest published chromosome-level genome of *O. kokonoricus* (*O. kokonorica*) [23], we acquired 77,758 transcripts in total. After a meticulous screening, 1624 isoforms originating from 1496 gene loci were considered as the lncRNA transcripts in *Orinus* for further investigation (Figure 1a and Supplementary Table S1). A total of 1624 lncRNAs were classified into several types based on their positional relationship with annotated genes in the reference assembly, including 1119 intergenic lncRNAs (u), 200 antisense lncRNAs (x), five intronic lncRNAs (i), and 300 sense lncRNAs (j and o) (Figure 1b). It was evident that although multiple types of lncRNAs exist in *Orinus*, intergenic lncRNAs (lincRNAs) were the major component in *Orinus*. A circos diagram consisting of 20 chromosomes depicted the basic features of *Orinus* lncRNAs at the transcriptome-wide level, containing the lncRNA classification and basic sequence characteristics of nucleic acids (Figure 1c). The number of lncRNAs located on the positive strand was 461, closely matching the number found on the negative strand, which was 504. The strand orientation of 658 lncRNAs remained unannotated. Furthermore, the length of the lncRNAs fell between 201 and 3976 nt, with an average of 644 nt (Figure 1d and Supplementary Table S2). lncRNAs exhibited significant variation in GC content, ranging from 0.2667 to 0.7283, with an average of 0.42 (Figure 1c). Additionally, the exon count of lncRNAs displayed diversity, with a majority of transcripts containing five or fewer exons. Specifically, 752 *Orinus* lncRNA transcripts (44.643%) comprised only one exon (Figure 1e and Supplementary Table S3). The fundamental characteristics of lncRNAs in *Orinus* leaves were largely consistent with those observed in other plants, thereby bridging the information gap on lncRNAs and facilitating a deeper understanding of the molecular and biological functions of non-coding genes in *Orinus*.

### 3.2. The Limited Sequence Conservation of lncRNAs in Orinus Implies That Species-Specific Noncoding RNAs Might Contribute to Speciation Adaptation Evolution

**Figure 2:**
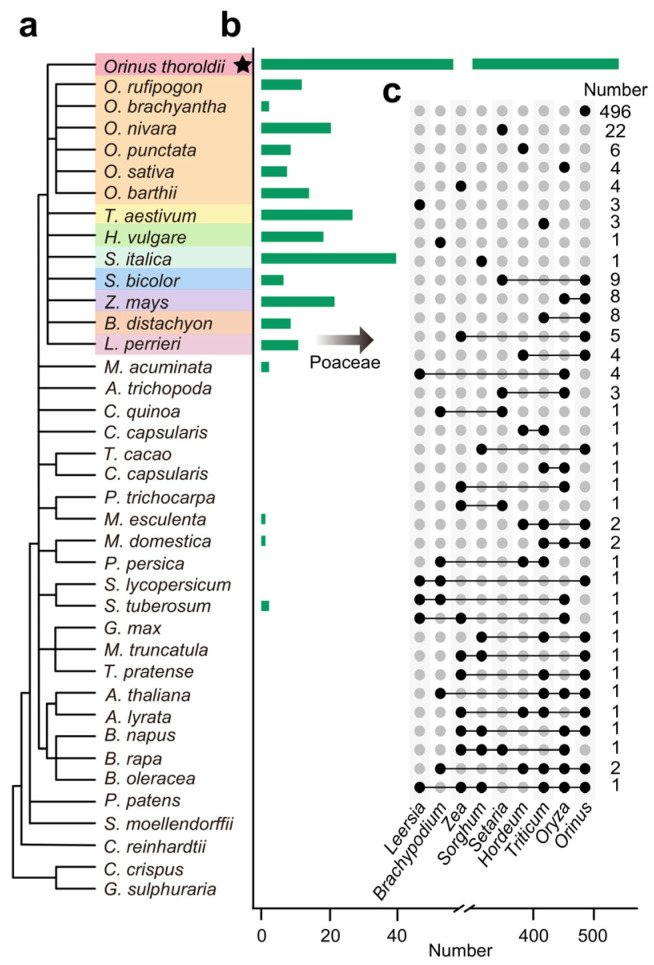
Contrast in the conservation of lncRNAs between *O. kokonoricus* and the other 39 plant species. (**a**) Illustrating the phylogenetic relationships of 39 plants, with Poaceae highlighted using colored segments. Asterisks denote the homologous or identical lncRNAs shared between *O. thoroldii* and *O. kokonoricus*. (**b**) The green bar chart illustrates the number of lncRNAs in each surveyed plant that are homologous to those in *O. kokonoricus*. (**c**) The unique and shared lncRNAs in nine Poaceae that are homologous to those in *O. kokonoricus*.

To investigate the sequence conservation of lncRNAs in *Orinus* comprehensively, we compared the evolutionary relationships of *Orinus* lncRNAs among 39 plant species (Figure 2a,b). Using *O. kokonoricus* as a reference species, we gained 627 lncRNAs (38.608% of the total) that could be homologous among the examined species. Among the nine subfamilies of Poaceae, sequence similarity was observed, with 546 (33.621%), 66 (4.064%), 25 (1.539%), 18 (1.108%), 38 (2.240%), seven (0.431%), 21 (1.293%), nine (0.554%), and 11 (0.677%). lncRNAs in *Orinus*, *Oryza*, *Triticum*, *Hordeum*, *Setaria*, *Sorghum*, *Zea*, *Brachypodium*, and *Leersia*, respectively, showing homology with *O. kokonoricus* (Figure 2b,c and Supplementary Table S4). It is a remarkable fact that we used the *O. kokonoricus* genome as the reference genome to conduct transcriptome mapping of *O. thoroldii*. Consequently, we found that 546 lncRNAs were the same lncRNAs between *O. thoroldii* and *O. kokonoricus* (Figure 2 and Supplementary Table S5). In non-Poaceae subfamilies or species, the highest homology percentage detected was 0.123%, which indicated that lncRNAs possessed lower sequence conservation. Combined with the poor sequence conservation of lncRNAs in *Orinus* and its unique growth environment, these *Orinus*-specific lncRNAs are likely to play a role in speciation adaptation evolution. 

### 3.3. DElncRNAs from Two Species in Orinus Provide Evidence for the Analysis of Speciation within the Genus

**Figure 3:**
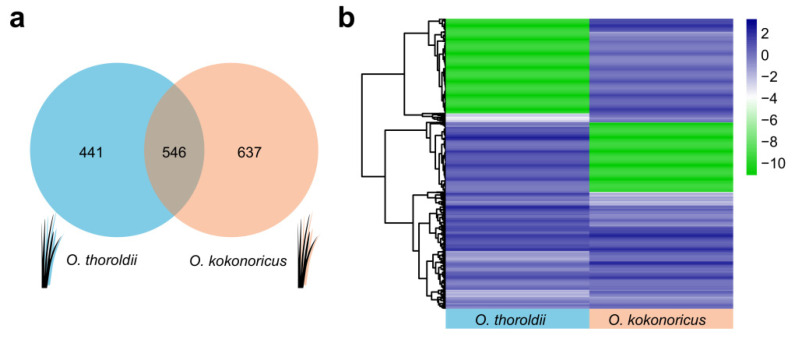
Expression pattern analysis of lncRNAs expressed in *O. thoroldii* and *O. kokonoricus*. (**a**) A Venn diagram was employed to illustrate the unique and shared genes expressed in *O. thoroldii* and *O. kokonoricus*. (**b**) Expression heatmap and hierarchical clustering of *O. thoroldii* and *O. kokonoricus* lncRNAs.

To further elucidate the differences between the two plants in *Orinus* and the molecular and evolutionary biological implications behind them, we conducted a preliminary comparison of lncRNAs in *O. kokonoricus* and *O. thoroldii*. Supplementary Table S7 lists all the FPKM values of a total of 1624 lncRNAs. The number of lncRNAs in *O. kokonoricus* was slightly higher than that in *O. thoroldii*, and the two species shared 546 lncRNAs. However, there were 441 species-specific *O. thoroldii* lncRNAs, and *O. kokonoricus* presented 637 unique lncRNAs (Figure 3a and Supplementary Table S6). In addition, we identified significant differentially expressed lncRNAs (DElncRNAs) between *O. thoroldii* and *O. kokonoricus*. To characterize DElncRNAs, we applied the following rigorous criterion: |log2 (fold change)| values ≥ 1, *p*-value ≤ 0.01. Based on the outcomes of the differential expression analysis with stringent criteria, we identified 286 DElncRNAs in total between *O. thoroldii* and *O. kokonoricus* (Figure 3b and Supplementary Table S8). Based on the preliminary comparison of lncRNAs from two ecologically and biologically distinct species in *Orinus*, species-specific DElncRNAs may serve as the molecular basis to explain the above differences.

### 3.4. Through Potential Cis-Regulation Patterns, DElncRNAs, and Their Target PCGs in Orinus Participate in Different Biological Processes

**Figure 4:**
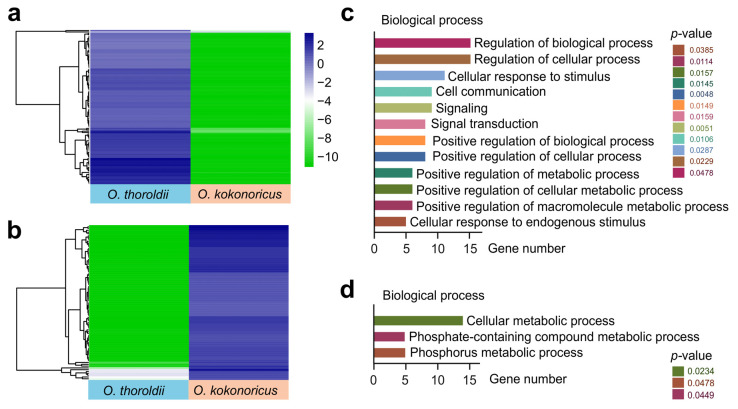
Expression patterns and function for potential *cis*-regulated target genes of DElncRNAs. (**a**) Expression heatmap and hierarchical clustering of cis-target genes expressed higher in *O. thoroldii* than *O. kokonoricus*. (**b**) Expression heatmap and hierarchical clustering of *cis*-target genes expressed higher in *O. kokonoricus* than in *O. thoroldii*. (**c**) Potential biological function enrichment of *O. thoroldii*. (**d**) Potential biological function enrichment of *O. kokonoricus*.

Based on the key role of lncRNA in the regulation of gene expression and the unexplored potential functions of lncRNA in *Orinus*, identifying and analyzing its target PCGs could help to understand the molecular identity and biological significance of lncRNA. To investigate the *cis*-regulation functions of these 286 DElncRNAs, we analyzed the expression patterns of their target PCGs located within a 100 kilobase (kb) window upstream and downstream of the DElncRNAs, in accordance with the transcriptome and genome of *Orinus*. We considered the adjacent PCGs exhibiting similar expression patterns to DElncRNAs as potential target genes and found 128 lncRNA-PCG pairs in *O. thoroldii* (77 lncRNAs and 113 PGCs) and 92 lncRNA-PCG pairs in *O. kokonoricus* (67 lncRNAs and 85 PCGs) (Supplementary Table S9). The heat map of target PCGs clearly demonstrated the differences in transcript levels between *O. thoroldii* and *O. kokonoricus* (Supplementary Table S10). In addition, through the information in the GO database, we recovered the potential functions of the target PCGs. In *O. thoroldii*, we identified a total of 19 lncRNA-PCG pairs (15 lncRNAs and 17 PCGs) through GO analysis, which were enriched in the “positive regulation of biological processes”, “metabolic processes”, the “cellular response to stimulus”, “signal transduction”, “cell communication”, and so on (Figure 4c). Meanwhile, in *O. kokonoricus*, we obtained 14 lncRNA-PCG pairs (13 lncRNAs and 14 PCGs) associated with “phosphate metabolism processes”, “cellular metabolism processes”, and “cellular metabolic processes” (Figure 4d). The different GO terms of the potential target PCGs of lncRNAs suggested that lncRNAs may regulate different biological processes and participate in specific adaptive evolutionary events in these two *Orinus* plants.

### 3.5. Detailed Investigation of Potential LncRNA Target Genes Provides Further Understanding of Speciation Adaptation about O. thoroldii

**Figure 5:**
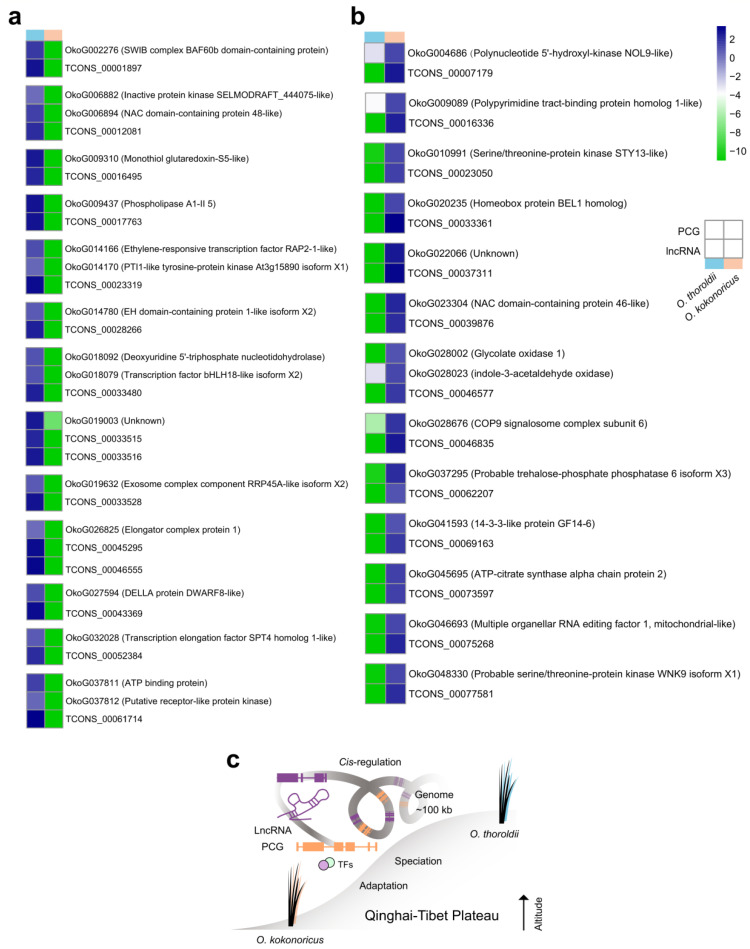
Potential biological functions of lncRNA-PCG pairs in two species of *Orinus*. (**a**) Expression of lncRNAs and their cis-regulated target genes in *O. thoroldii* and their functional annotation. (**b**) Expression of lncRNAs and their cis-regulated target genes in *O. kokonoricus* and their functional annotation. (**c**) In the QTP, lncRNA participates in the *cis*-regulation model of *Orinus* speciation adaptation.

To understand the function of PCGs near the lncRNA genomic locus, we used BLASTP to examine the basic information on PCGs in detail (Figure 5). In *O. thoroldii*, the target PCG of TCONS_00012081 (OkoG006894) was the NAC domain-containing protein 48-like associated with plant stress tolerance. OkoG009437 (phospholipase A1-II 5) was the target PCG that played an important role in the abiotic stresses. In addition, OkoG014166, ethylene-responsive transcription factor RAP2-1-like, could co-express with TCONS_00023319 and participate in hypoxia survival. Moreover, TCONS_00033480 targeted OkoG018079 and encoded the transcription factor bHLH18-like isoform X2, which could function in stress responses. OkoG027594 was the target PCG of TCONS_00043359 encoding DELLA protein DWARF8-like, which is involved in the crux of many plant developmental pathways. TCONS_00061714 targeted OkoG037811, a putative receptor-like protein kinase that can participate in signal transduction processes as receptors, regulating plant responses to stress in different plants. A series of target PCGs of lncRNAs in *O. kokonoricus* were associated with regulating basic growth and development processes, photosynthesis, and the production of various metabolites in plants. Among them, one target PCG OkoG023304 of TCONS_00039876 encoding NAC domain-containing protein 46-like related to plant stress resistance (Figure 5b). In summary, some of the lncRNAs identified in *O. thoroldii* have the potential to contribute to plant defense and stress resistance. In addition, to clarify the role of TFs in *Orinus*, we found three PCGs with TF functions in *O. thoroldii* including the ATAF, NAM, and CUC families (NACs), basic helix-loop-helices (bHLHs), and the GAI, RGA, and SCR families (GRASs) (Supplementary Table S11). It is noteworthy that the expression events of TFs were exclusive to *O. thoroldii*, suggesting that these DElncRNAs may aid *O. thoroldii* in adapting to the QTP environment through the expression regulation of TFs.

## 4. Discussion

Transcriptome analyses have revealed that substantial portions of eukaryotic genomic sequences are transcribed to lncRNAs [39]. The prospective functions of lncRNAs have recently come to light, with a growing focus on their role in regulating plant development and resistance. A considerable number of lncRNAs are linked to plant defense mechanisms [40,41,42]. Yet, there is limited research on how lncRNAs mediate the speciation adaptation in plants. For this reason, substantial endeavors should be dedicated to grasping the physiological roles of lncRNAs between *O. thoroldii* and *O. kokonoricus*.

A previous study showed that the *Orinus* genus consists of two main species including *O. thoroldii*, which is predominantly distributed in the western QTP at elevations between 3300 and 4700 m, and *O. kokonoricus*, in the eastern QTP at a relatively low altitude between 2500 and 3400 m [22]. In order to better understand the roles of lncRNAs on the adaption of *Orinus* in the QTP, we performed a systematic bioinformatics analysis based on previously published leaf transcriptome data of *O. thoroldii* and *O. kokonoricus*, which were collected at Gongga country in Xizang at the elevation of 4687 m and Gonghe country in Qinghai at the elevation of 3130 m [27]. After a rigorous transcript screening pipeline and differential expression analysis, we finally identified 286 DElncRNAs between *O. thoroldii* and *O. kokonoricus* from a total of 1624 lncRNA transcripts in *Orinus* leaves, providing a molecular basis for distinguishing these two geographically isolated species in *Orinus.*

In animals and plants, only a small part of lncRNAs (approximately 2–2.5%) had evolutionary conservation in primary sequences, and even lncRNAs with regulatory functions also show rapid sequence evolution [43,44,45]. However, we compared these with lncRNAs of 39 plant species and found that lncRNAs in *Orinus* were conserved in the Poaceae family. Past research suggested that lncRNA is less conserved than mRNA [46]; however, there was still some evolutionary sequence conservation among allied species, which may be related to the fact that they have similar functions. This result agrees well with previous studies [47]. The diverse environmental conditions of the QTP influenced the rapid ecological isolation of species (such as *Orinus*) and subsequent divergence and adaptive evolution. Compared with plants in other geographical locations and evolutionary processes, the lncRNAs with low sequence conservation in *Orinus* were most likely to be molecular manifestations of speciation adaptation.

As previously described, lncRNAs play crucial roles in both upregulating and downregulating gene expression in plants and mammals. They are essential in processes like differentiation, environmental adaptation, and so on [48]. In the nucleus, the ability of lncRNAs to regulate gene expression, either by acting in cis on adjacent genes [49,50] or acting in trans regardless of gene location [51,52,53]. One example is a lncRNA-CIL1 in *Arabidopsis thaliana*, which responds to cold stress by affecting the reactive oxygen species pathway or osmotic regulation substances. A transcription analysis revealed that CIL1 regulates the expression of downstream cold stress response genes, enabling plants to respond accordingly [54]. Therefore, understanding the underlying mechanism between *O. thoroldii* and *O. kokonoricus* is important. Based on the above, we indirectly predicted the function of DElncRNAs by predicting *cis*-target PCGs. Eventually, we recognized 128 lncRNA-PCG pairs, composed of 77 lncRNAs and 113 PCGs in *O. thoroldii*, whereas 92 lncRNA-PCG pairs comprised 67 lncRNAs and 85 PCGs in *O. kokonoricus*. Furthermore, the Gene Ontology analysis of the protein sequences encoded by these target genes found that the PCGs of *O. thoroldii* were enriched in the regulation of biological functions, whereas the PCGs of *O. kokonoricus* were enriched in the model of phosphate and phosphate-containing compound metabolic processes. It was intriguing that these two *Orinus* species of PCGs were enriched in serving the function of significantly diverse biological processes. We speculate that the distinct altitudinal distributions of the two species of *Orinus* (the distribution altitude of *O. thoroldii* is approximately 1000 m higher than that of *O. kokonoricus*) have led to completely different processes of enrichment, which will be a key factor in uncovering their adaptability to their environments. Although we obtained general understanding of the function of these lncRNAs through target gene annotation, the intrinsic mechanism of action is ambiguous and requires a great deal of follow-up work. 

More importantly, we found three TFs in *O. thoroldii* that were family members of NACs, bHLHs, and GRASs. In addition, NAC TFs were found to have a prominent role in mediating plant responses to abiotic stresses [55,56,57]. In tobacco, a novel NAC TF (*NtNAC053a*) was found to enhance drought and salt tolerance [58]. Moreover, bHLH TFs were not only widely implicated in plant growth but also served this function in plant responses to abiotic stress [59]. *AhbHLH112* acted as a positive factor in drought stress tolerance, and *ZmPTF1*, a bHLH family member, regulated drought tolerance in maize by facilitating root growth and abscisic acid synthesis [60,61]. Also, Ma et al. [62] demonstrated that a member of GRAS TFs, *PeSCL7*, was potentially useful for engineering drought- and salt-tolerant trees. However, the overexpression of *SlGRAS7* can result in conferring abiotic stress tolerance in tomatoes [63]. Therefore, we recommend focusing on these three TFs predicted in *O. thoroldii* because they can be the key point to understanding speciation adaptation in two species of *Orinus*. Our work linked lncRNAs to PCGs near their genomic sites and discovered a series of key factors (especially TFs), providing a molecular model for further elucidating speciation and adaptation in *Orinus* from the perspective of non-coding genes (Figure 5c). These results suggested that the detailed mechanism underlying how lncRNAs respond to abiotic stresses in *O. thoroldii* and *O. kokonoricus* requires further study down the road.

## 5. Conclusions

This study systematically identified 1624 lncRNAs and characterized their transcriptome-wide features in two species of *Orinus* including *O. thoroldii* and *O. kokonoricus*. LncRNA screening set the foundation for the further understanding of the molecular and biological functions of non-coding genes in *Orinus*. According to the evolutionary tree implicating the relationships among the 39 species with the count of conserved lncRNAs, the limited sequence conservation of lncRNAs in *Orinus* implied that specific noncoding RNAs might contribute to speciation adaptation evolution. DElncRNAs from two species in *Orinus* also provided evidence for the analysis of speciation within the genus. Furthermore, we analyzed the expression data of DElncRNAs with their nearby protein-coding genes between *O. thoroldii* and *O. kokonoricus*. Through potential *cis*-regulation patterns, lncRNAs of different *Orinus* and their target PCGs could participate in different biological processes, which were associated with environmental adaption and involved in abiotic stress responses. These results provided a fresh perspective on the evolutionary adjustments of these species to various ecological environments on the QTP and the mechanism behind the disparities observed in *O. thoroldii* and *O. kokonoricus*. In short, the lncRNAs discovered in *Orinus* enhanced our understanding of the lncRNAs implicated in the speciation adaptation in *Orinus* and offered a wealth of resources for upcoming comparative and functional investigations.

## Data Availability

Data availability is not applicable to this article as no new data were created or analyzed in this study.

## Supplementary Materials

The following supporting information can be downloaded at: https://www.mdpi.com/article/10.3390/biology13050349/s1, Table S1. Transcriptome-wide information on 1624 long noncoding RNA (lncRNA) transcripts in two species of *Orinus*. Table S2. Length distribution of lncRNAs in two species of *Orinus*. Table S3. The exon number of lncRNAs in two species of *Orinus*. Table S4. The number of homologous lncRNAs in *O. kokonoricus* compared with 39 other species. Table S5. Homologous lncRNAs in *O. kokonoricus* compared with 39 other species. Table S6. Unique and shared genes expressed in two species of *Orinus*. Table S7. FPKM values of 1624 lncRNAs in two species of *Orinus*. Table S8. FPKM values of DElncRNAs in two species of *Orinus*. Table S9. *Cis*-regulated target PCGs and their DElncRNAs. Table S10. FPKM values of *cis*-regulated target PCGs of DElncRNAs in two species of *Orinus*. Table S11. Expression pattern of *cis*-regulated TFs and their DElncRNAs in the two species of *Orinus*.
